# Synthesis, Biological Activities, and Quantitative Structure–Activity Relationship (QSAR) Study of Novel Camptothecin Analogues

**DOI:** 10.3390/molecules20058634

**Published:** 2015-05-13

**Authors:** Dan Wu, Shao-Yong Zhang, Ying-Qian Liu, Xiao-Bing Wu, Gao-Xiang Zhu, Yan Zhang, Wei Wei, Huan-Xiang Liu, An-Liang Chen

**Affiliations:** 1 Local and National Joint Engineering Laboratory of Biopesticide High-Efficient Preparation Technology, Zhejiang A&F University, Lin’an 311300, China; E-Mails: wud2012@lzu.edu.cn (D.W.); 1zhangshaoyong@163.com (S.-Y.Z.); wuxb13@lzu.edu.cn (X.-B.W.); 2 School of Pharmacy, Lanzhou University, Lanzhou 730000, China; E-Mails: zhugx13@lzu.edu.cn (G.-X.Z.); Zhangyan10@lzu.edu.cn (Y.Z.); WeiW13@lzu.edu.cn (W.W.)

**Keywords:** camptothecin, synthesis, biological activity, SAR analysis, CoMFA

## Abstract

In continuation of our program aimed at the development of natural product-based pesticidal agents, three series of novel camptothecin derivatives were designed, synthesized, and evaluated for their biological activities against *T. Cinnabarinus*, *B. brassicae*, and *B. xylophilus*. All of the derivatives showed good-to-excellent activity against three insect species tested, with LC_50_ values ranging from 0.00761 to 0.35496 mmol/L. Remarkably, all of the compounds were more potent than CPT against *T. Cinnabarinus*, and compounds **4d** and **4c** displayed superior activity (LC_50_ 0.00761 mmol/L and 0.00942 mmol/L, respectively) compared with CPT (LC_50_ 0.19719 mmol/L) against *T. Cinnabarinus*. Based on the observed bioactivities, preliminary structure–activity relationship (SAR) correlations were also discussed. Furthermore, a three-dimensional quantitative structure–activity relationship (3D-QSAR) model using comparative molecular field analysis (CoMFA) was built. The model gave statistically significant results with the cross-validated q^2^ values of 0.580 and correlation coefficient r^2^ of 0.991 and $r_{\mathrm{pred}}^{2}$ of 0.993. The QSAR analysis indicated that the size of the substituents play an important in the activity of 7-modified camptothecin derivatives. These findings will pave the way for further design, structural optimization, and development of camptothecin-derived compounds as pesticidal agents.

## 1. Introduction

The application of chemical pesticides in agriculture worldwide is still the most widespread method for today’s insect pest management. However, rising resistance to available agrochemicals, combined with their adverse side effects on the environment and human health, is driving the search for new alternative molecules to some existing commercial products [1,2,3]. It is well recognized that plant secondary metabolites result from the interaction between plants and the environment during the long period of evolution in plants, and pesticides produced from plant secondary metabolites may result in less or slower resistance development and lower pollution [4,5,6]. Intriguingly, some botanical pesticides, such as pyrethroids, rotenones, and neonicotinoids, which emerged as attractive alternatives to traditional chemical pesticides and had been widely commercialized, now have their own prominence [7]. Hence, the discovery of new pesticidal compounds directly from plant secondary metabolites or by using them as the lead compounds for further modification and structure optimization has recently been important in the research and development of new pesticides [8,9].

**Figure 1:**
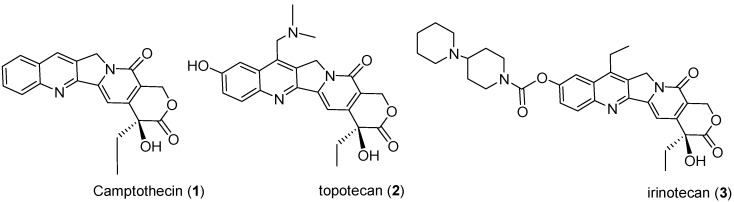
Chemical structures of camptothecin (**1**), topotecan (**2**), and irinotecan (**3**).

Camptothecin (CPT, **1**, Figure 1), a naturally occurring quinoline alkaloid, is the main secondary metabolite isolated from the wood and bark of the Chinese tree *C. acuminate*. Besides its use as the lead compound for the preparation of potent anticancer drugs, such as topotecan (**2**) and irinotecan (**3**) [10,11], it also received much research attention for its interesting pesticidal activities [12,13,14]. In ancient China, the crude extract of *C. acuminate* containing CPT has been used traditionally to control insect pests for centuries, and it was reported to be a potent chemosterilant against the housefly and cabbage caterpillar [15,16]. It also exhibited significant toxic effect against *Empoasca vitis*, *Nilaparvata lugens*, and *Chilo suppressalis*, which suggested its potential application as an insecticide in the field [17]. Additionally, a recent study showed that CPT could cause visible changes in the midgut of the lepidopteran pests *Trichoplusia ni* and *Spodoptera exigua*, such as losing the single layer of epithelial cells and disrupting the peritrophic membrane [18]. Investigations by Zhong et al. also demonstrated that CPT-induced apoptosis in SL-1 cells and midgut cells of *S. litura* [19]. Consistent with these results, Zhang *et al*. recently revealed that CPT caused Sf21 and IOZCAS-Spex-II cell apoptosis via a mitochondrial-dependent apoptosis signal transduction pathway [20], suggesting that its mode of action may be related to apoptosis. Moreover, pretreatment with CPT led to reduction in both the enzymatic activity and the steady accumulation of the Topo I protein in IOZCAS-Spex-II cells despite up-regulation of its mRNA expression in response to the treatment [21]. In connection with these efforts, in order to find new camptothecin-derived insecticides with improved profiles and to clarify the structure–activity relationships of camptothecin analogues, a number of CPT derivatives modified in the different positions have been synthesized and their insecticidal activity evaluated against some important insect pests by our group [22,23,24,25,26,27]; among which, some compounsd exhibited insecticidal activity equal to or higher than that of toosendanin, a commercial botanical insecticide isolated from *Melia azedarach*. Especially, some C-7 substituted CPT derivatives showed promising insecticidal activity against *B. longissima*, *B. xylophilus*, and *T. cinnabarinus* [25,26,27], suggesting the possibility of further optimizing CPT through rational C-7 modifications.

**Scheme 1:**
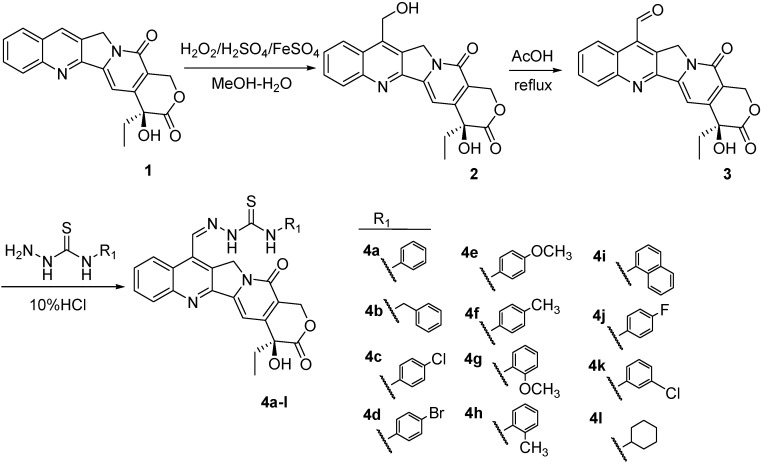
Synthesis of 7-(*N*-substituted-thioureidohydrazono)-formyl-camptothecin (**4a**–**l**).

**Scheme 2:**
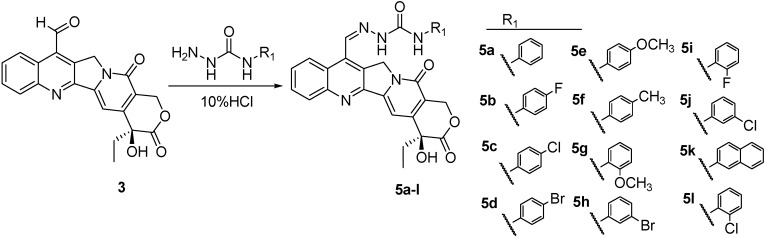
Synthesis of 7-(*N*-substituted-carbamidohydrazono)-formyl-camptothecin (**5a**–**l**).

**Scheme 3:**
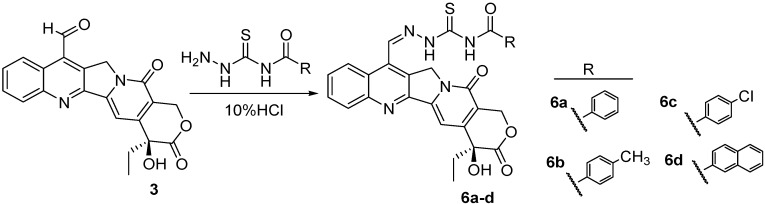
Synthesis of 7-(*N*-acylsubstituted-thioureidohydrazono)-formyl-camptothecin (**6a**–**d**).

These encouraging results prompted us to further extend our investigation by incorporating three functional fragments (*i.e.*, urea, thiourea, and acylthiourea) into C-7 substituted CPT and synthesizing three series of novel CPT derivatives. As is well known in crop protection and bioactive chemicals, ureas, thioureas, and acylthioureas have been reported to display a variety of biological activities, such as insecticidal, fungicidal, herbicidal, antimicrobial, antitumor, *etc.* [28,29,30,31,32,33], Furthermore, the hydrazone group is identified as an important pharmacophore that is widely used in pesticide and drug molecular design [34,35,36,37]. With this in mind, in this paper we incorporated three functional fragments into CPT at C-7 position through hydrazone as linker and synthesized three series of novel CPT derivatives according to Scheme 1, Scheme 2 and Scheme 3. Their lethal activities against *T. Cinnabarinus*, *B. brassicae*, and *B. xylophilus* were tested, and the median lethal concentrations (LC_50_) were calculated accordingly. Based on the observed bioactivities, the structure–activity relationship (SAR) of these analogs was also discussed. Furthermore, a three-dimensional quantitative structure–activity relationship (3D-QSAR) model using comparative molecular field analysis (CoMFA) was built to understand the deep relationship between the biological activity and molecular structure of CPT analogues.

## 2. Results and Discussion

### 2.1. Chemistry

The synthetic routes to target camptothecin derivatives **4a**–**l**, **5a**–**l** and **6a**–**d** are outlined in Scheme 1, Scheme 2 and Scheme 3. Briefly, CPT was firstly converted into 7-hydroxymethyCPT (**2**) through the Minisci free radical reaction promoted by the aqueous acidic FeSO_4_/H_2_O_2_/CH_3_OH reagent system, and then refluxed with acetic acid to give the intermediate camptothecin-7-aldehyde **3**. Next, intermediate **3** was coupled with various carbonylhydrazones in CHCl_3_-EtOH to afford the desired compounds **4a**–**l**, **5a**–**l** and **6a**–**d**, respectively. All synthesized target compounds were purified by column chromatography, and their structures were characterized by m.p., IR, ^1^H-NMR, and elemental analysis.

### 2.2. Biological Activity

Based on the methodology in Scheme 1, Scheme 2 and Scheme 3, three series of new camptothecin derivatives (**4a**–**l**, **5a**–**l** and **6a**–**d**) were obtained and examined for their lethal activity against *T. Cinnabarinus*, *B. xylophilus*, and *B. brassicae*, and the median lethal concentrations (LC_50_) were calculated accordingly. CPT was included as control and the results are summarized in Table 1. In preliminary bioassays, all of the derivatives showed good-to-excellent activities against three insect species tested, with LC_50_ values ranging from 0.00761 to 0.35496 mM. Notably, all new compounds exhibited promising acaricidal activity against *T. Cinnabarinus*, with LC_50_ values ranging from 0.00761 to 0.16309 mM, and were more potent than CPT (LC_50_: 0.19719 mM), but they displayed less potency than avermectin (0.013 μM). Among them, compounds **4d** (LC_50_: 0.00761 mM) and **4c** (LC_50_: 0.00942 mM) exhibited the greatest acaricidal activity against *T. Cinnabarinus* in all derivatives we prepared, suggesting that C-7 modified camptothecin derivatives were more lethal against *T. Cinnabarinus* and this position may be amenable to further synthetic modification for lethal potency. Moreover, except for compound **6a** (LC_50_: 0.03649 mM), all other compounds showed similar or less potent nematicidal activities against *B. xylophilus* with LC_50_ values of 0.07944–0.42748 mM compared to that of CPT (LC_50_: 0.06861 mM). Meanwhile, against *B. brassicae*, most compounds were somewhat more active than that of CPT. In particular, **4b** and **4d** displayed the best promising lethal activity, with LC_50_ values of 0.05939 and 0.05943 mM, respectively.

**Table 1:** Biological activity of compounds **4a**–**l**, **5a**–**l** and **6a**–**d** against *T. Cinnabarinus*, *B. xylophilus*, and *B. brassicae* (Linnaeus).

Compd	LC_50_ (mM)			
	*Tetranychus cinnabarinus*	*Bursaphelenchus xylophilus*	*Brevicoryne brassicae*
**4a**	0.09611	0.26150	0.20589	
**4b**	0.02384	0.16486	0.05939	
**4c**	0.00942	0.11331	0.15621	
**4d**	0.00761	0.27035	0.05943	
**4e**	0.07299	0.24100	0.35496	
**4f**	0.09457	0.24809	0.17584	
**4g**	0.13990	0.22729	0.11500	
**4h**	0.01156	0.24162	0.08253	
**4i**	0.01841	0.13670	0.20292	
**4j**	0.08835	0.28470	0.12832	
**4k**	0.06152	0.09212	0.15807	
**4l**	0.08218	0.26553	0.18599	
**5a**	0.04029	0.29812	0.11830	
**5b**	0.05929	0.07944	0.20210	
**5c**	0.08796	0.36781	0.21269	
**5d**	0.01707	0.20740	0.16198	
**5e**	0.06648	0.12162	0.10958	
**5f**	0.16309	0.42748	0.40716	
**5g**	0.04171	0.20948	0.17947	
**5h**	0.05408	0.18991	0.15555	
**5i**	0.05913	0.24922	0.12422	
**5j**	0.06063	0.10277	0.19429	
**5k**	0.08516	0.11697	0.15819	
**5l**	0.02745	0.21472	0.08071	
**6a**	0.09915	0.03649	0.11815	
**6b**	0.05079	0.32122	0.11710	
**6c**	0.03930	0.30762	0.07464	
**6d**	0.04877	0.27567	0.11019	
**1**	0.19719	0.06861	0.14593	

As shown in Table 1, against *T. cinnabarinus*, within thiourea side chains, active compounds with a *p*-bromophenyl moiety (**4d**, LC_50_: 0.00761 mM) were generally slightly more potent than corresponding analogs with a *p*-chlorophenyl or *p*-fluorophenyl group (**4c** and **4j**). Changing the position of the chloro substituent from C-4 in phenyl ring to C-3 in **4c** and **4k** affected the potency. Lethal potency for compounds **4a**–**l** was slightly affected by the electronegativity or positions of substituents on the phenyl ring, but the differences in potency were often not large. In contrast, investigation of different side chains **5a**–**l** and **6a**–**d** gave similar interesting results, and the order of potency was somehow changed; further investigation is needed. When compounds **4c** and **4d** are compared to compounds **5c** (LC_50_: 0.08796) and **5d** (LC_50_: 0.01707), we could find that one atom difference between thiourea and urea can cause a change in their lethal activity.

In addition, the LC_50_ rates of the target compounds against *T. Cinnabarinus* formed a sharp contrast to that of the activities against the other two insect species, but gave similar structure–activity relationships for each species. Taken together, this study showed that three series of camptothecin derivatives could exert significant insecticidal/acaricidal activity and changes in the substituents could lead to remarkable changes in potency. Simultaneously, these results clearly underlined that the lethal differences could be ascribed to a combination of factors, like the nature of the substitutes (which may depend on the size of substitutes, electronic characteristics of substitutes, or other factors) or a different interaction at the site.

### 2.3. CoMFA Analysis

**Figure 2:**
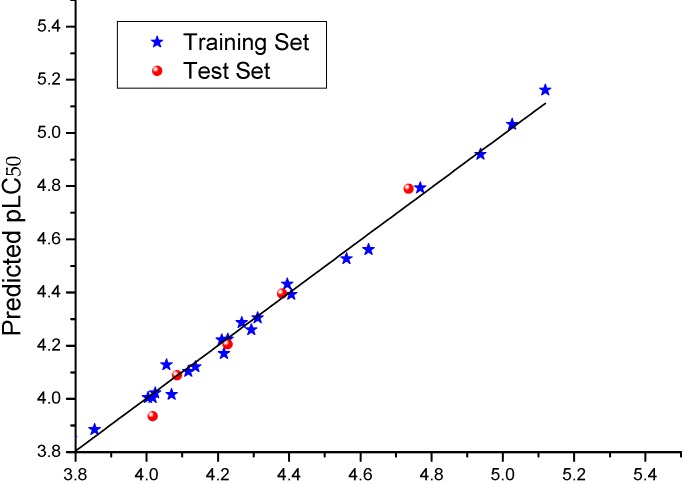
The correlation plot of the experimental activities *versus* predicted activities of the studied compounds based on the model of COMFA.

**Figure 3:**
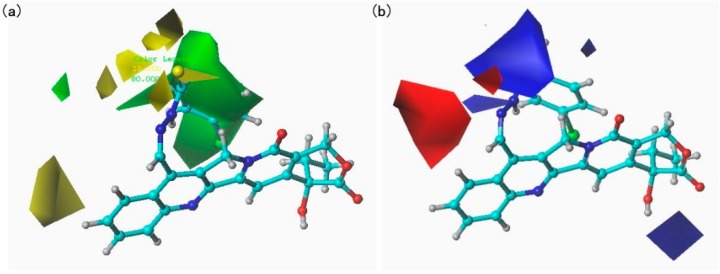
CoMFA StDev * Coeff contour maps based on compounds **4d**. (**a**) Steric field: green contours (80% contribution), yellow contours (20% contribution). (**b**) Electrostatic field: blue contours (80% contribution), red contours (20% contribution).

The results of CoMFA analysis are listed in Table 2 and Table 3. The model gave a cross-validated *q*^2^ of 0.580 with six components and non-cross-validated r^2^ of 0.991 with *SEE* of 0.041, showing that the built model had a good conventional statistical correlation and high predictive ability. The correlation between the predicted and experimental activities was plotted in Figure 2. To further validate the predictive ability of the obtained model, five compounds not included in the construction of CoMFA model were used as the test set. The predicted results of the test set were also listed in Table 3 (asterisk labeled) and shown in Figure 2. From Figure 2, it can be seen clearly that the predicted pLC_50_ values of the test set compounds are in good agreement with the experimental data, with $r_{\mathrm{pred}}^{2}$ of 0.993, indicating that the CoMFA model could be reliably used to predict the activity of new compounds and design novel inhibitors. The final non-cross-validated partial least squares analysis was further used to generate 3D contour maps for CoMFA using the field type stdev*coeff. The contour maps (shown in Figure 3) can provide a more detailed understanding of the key structural features required for the biological activity. Figure 3a,b shows the contours of the steric and electrostatic maps, respectively. Here, the contributions of the steric field (0.709) are twice those of the electrostatic field (0.291), indicating that the effects of the steric field on the model are larger than the electrostatic field.

**Table 2:** PLS statistics of the CoMFA.

Cross-validation		No cross-validation	
q^2^	component	r^2^	SEE
0.580	6	0.991	0.041

**Table 3:** Results of experimental and predicted pLC_50_.

Compd	Act.	Pred.	Res.	Compd	Act.	Pred.	Res.
*4a	4.017	3.936	−0.081	**5c**	4.056	4.128	0.072
**4b**	4.623	4.562	−0.061	**5d**	4.768	4.794	0.026
**4c**	5.026	5.032	0.006	**5e**	4.117	4.104	−0.013
**4d**	5.119	5.161	0.042	**5f**	3.788	3.852	0.064
**4e**	4.137	4.121	−0.016	*5g	4.380	4.397	−0.059
**4f**	4.024	4.022	−0.002	**5h**	4.267	4.287	0.020
**4g**	3.854	3.885	0.031	**5i**	4.228	4.225	−0.003
**4h**	4.937	4.920	−0.017	**5j**	4.217	4.170	−0.047
*4i	4.735	4.791	0.056	**5k**	4.070	4.017	−0.053
**4j**	4.017	4.005	−0.012	**5l**	4.561	4.527	−0.034
**4k**	4.211	4.222	0.011	**6a**	4.004	4.005	0.001
*4l	4.085	4.089	0.004	**6b**	4.294	4.260	−0.034
**5a**	4.395	4.432	0.037	**6c**	4.406	4.393	−0.013
*5b	4.227	4.206	−0.021	**6d**	4.312	4.305	−0.007

**Figure 4:**
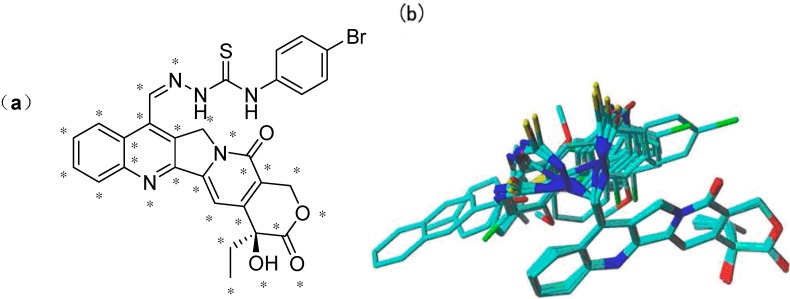
(**a**) The common scaffold (marked atoms with star) of camptothecin derivatives in the alignment shown in compound **4d**; (**b**) the alignment of camptothecin derivatives using the low energy conformers by atom fit.

In the following analysis of CoMFA contour maps, compound **4d** was selected as the reference molecule. In the contour map of the steric field, the green contours indicate regions where large substituents are favorable for the inhibitory activity, whereas the yellow contours indicate regions where large substituents are unfavorable for activity. In the contour map of the electrostatic field, the blue contours indicate regions where electropositive substituents increase activity, whereas the red contours indicate regions where electronegative substituents increase activity. As shown in Figure 4a, a large green contour around the p-bromophenyl group of compound **4d** was recognized, indicating that large substituents in the position are favorable for the inhibitory activity. For example, the order of the activities of 7-modified camptothecin derivatives containing thiourea moieties is **4d** > **4c** > **4j**. In a series of derivatives containing urea moieties, the activity of compound **5d** are higher than compound **5c**. In a series of 7-modified camptothecin derivatives containing acylthiourea moieties, compound **6c** is more favorable than compound **6a**. A large yellow contour is also observed around the A ring of compound **4d**. The observation is in accord with the experimental determinations such as **5e** < **5b** < **5a**.

In the contour map of the electrostatic field (Figure 3b), a large blue contour and a large red contour appear near the sulfur atoms of compound **4d**, indicating that electrostatic contributions of thiourea, urea, and acylthiourea moieties in the position have little influence on activity. Another small blue contour is also observed around the E ring, which indicates that the introduction of electropositive substituents was favorable for activity. The developed QSAR model based on the above analysis was helpful in understanding the key structural factors affecting the bioactivity of this series of compounds.

## 3. Experimental Section

### 3.1. General

All reagents and solvents were of reagent grade or purified according to standard methods before use. Analytical thin-layer chromatography (TLC) and preparative thin-layer chromatography (PTLC) were performed with silica gel plates using silica gel 60 GF254 (Qingdao Haiyang Chemical Co., Ltd., Qingdao city, China). Melting points were determined in Kofler apparatus and were uncorrected. IR spectra were obtained on NIC-5DX spectra photometer, ^1^H-NMR spectra were recorded at 400 MHz on a Bruker AM-400 spectrometer using TMS as reference (Bruker Company, Boston, MA, USA). Elemental analyses were determined on a Vario El Gmbh elemental analyzer. The starting camptothecin was isolated from a Chinese medicinal plant *C. acuminata* and was purified before being used. A key intermediate for the synthesis of three series of target compounds was camptothecin-7-aldehyde (**3**), which was prepared according to Sawada *et al.* [38].

### 3.2. General Synthetic Procedure for Target Compounds **4a**–**l**

To a solution of 7-formyl camptothecin (0.1 mmol) in chloroform (10 mL) and methanol (10 mL), an appropriate *N*-substituent-thioureidohydrazine (0.12 mmol) and 10% hydrochloric (0.5 mL) were added. The reaction mixture was stirred for 2 h at room temperature. A large amount of precipitate was producted. After filteration, the precipitate was washed with chloroform to give a yellow solid. Recrystallization from *N*, *N*-dimethyl formamine gave compounds **4a**–**l**.

*7-(N-phenylthioureidoiminomethyl)-camptothecin* (**4a**). Yield: 85%; mp: 235 °C (decomp.); IR(KBr) cm^−1^: 3444 (OH), 3324, 3287 (N-H), 1737 (γ-lactone), 1655 (C=N), 1253 (C=S); ^1^H-NMR (400 MHz, DMSO-*d*_6_) δ: 0.86–0.90 (m, 3H, 19-H), 1.82–1.93 (m, 2H, 18-H), 5.44 (s, 2H, 5-H), 5.66–5.78 (m, 2H, 17-H), 6.54 (s, 1H, 20-OH), 7.22–7.26 (m, 1H, 4'-H), 7.36 (s, 1H, 14-H), 7.40–7.44 (m, 2H, 2', 6'-H), 7.82 (t, 1H, 11-H), 7.85–7.89 (m, 2H, 3', 5'-H), 7.92 (t, 1H, 10-H), 8.24 (d, *J =* 8.0 Hz, 1H, 12-H), 8.33 (d, *J =* 8.0 Hz, 1H, 9-H), 9.19 (s, 1H, 7-CH), 10.01 (s, 1H, CSNHC), 12.32 (s, 1H, NNH). Anal. calc. for C_28_H_23_N_5_O_4_S: C 63.99%, H 4.41%, N 13.33%. Found: C 63.98%, H 4.42%, N 13.34%.

*7-(N-benzylthioureidoiminomethyl)-camptothecin* (**4b**). Yield: 89%; mp: 243 °C (decomp.); IR(KBr) cm^−1^: 3436 (OH), 3387, 3266 (N-H), 1747 (γ-lactone), 1659 (C=N), 1258 (C=S); ^1^H-NMR (400 MHz, DMSO-*d*_6_) δ: 0.86–0.89 (m, 3H, 19-H), 1.84–1.91 (m, 2H, 18-H), 4.89 (d, *J =* 5.6 Hz, 2H, benzyl-CH_2_), 5.45 (s, 2H, 5-H), 5.57–5.73 (m, 2H, 17-H), 6.53 (s, 1H, 20-OH), 7.23–7.27 (m, 1H, 4'-H), 7.35 (s, 1H, 14-H), 7.38 (d, *J =* 7.2 Hz, 2H, 2', 6'-H), 7.45 (d, *J =* 7.2 Hz, 2H, 3', 5'-H), 7.79 (t, 1H, 11-H), 7.90 (t, 1H, 10-H), 8.21 (d, *J =* 8.4 Hz, 1H, 12-H), 8.32 (d, *J =* 8.4 Hz, 1H, 9-H), 9.07 (s, 1H, 7-CH), 9.08 (s, 1H, CSNHC), 12.05 (s, 1H, NNH). Anal. calc. for C_29_H_25_N_5_O_4_S: C 64.55%, H 4.67%, N 12.98%. Found: C 64.56%, H 4.66%, N 12.98%.

*7-[N-(4-chlorinephenyl)thioureidoiminomethyl]-camptothecin* (**4c**). Yield: 76%; mp: 265 °C (decomp.); IR(KBr) cm^−1^: 3437 (OH), 3321, 3273 (N-H), 1730 (γ-lactone), 1651(C=N), 1249(C=S); ^1^H-NMR (400 MHz, DMSO-*d*_6_): δ 0.86–0.89 (m, 3H, 19-H), 1.82–1.93 (m, 2H, 18-H), 5.44 (s, 2H, 5-H), 5.69–5.81 (m, 2H, 17-H), 6.55 (s, 1H, 20-OH), 7.37 (s, 1H, 14-H), 7.47 (d, *J =* 8.8Hz, 2H, 2', 6'-H), 7.83–7.87 (m, 1H, 11-H), 7.91 (d, *J =* 8.8Hz, 2H, 3', 5'-H), 7.94–7.96 (m, 1H, 10-H), 8.26 (d, *J =* 8.0 Hz, 1H, 12-H), 8.35 (d, *J =* 8.0 Hz, 1H, 9-H), 9.20 (s, 1H, 7-CH), 10.11 (s, 1H, CSNHC), 12.42 (s, 1H, NNH). Anal. calc. for C_28_H_22_N_5_O_4_SCl: C 60.05%, H 3.96%, N 12.51%. Found: C 60.07%, H 3.94%, N 12.51%.

*7-[N-(4-bromophenyl)-thioureidoiminomethyl]-camptothecin* (**4d**). Yield: 83%; mp: 253 °C (decomp.); IR(KBr) cm^−1^: 3436 (OH), 3321, 3266 (N-H), 1729 (γ-lactone), 1650 (C=N), 1248 (C=S); ^1^H-NMR (400 MHz, DMSO-*d*_6_) δ: 0.86–0.89 (m, 3H, 19-H), 1.82–1.93 (m, 2H, 18-H), 2.33 (s, 3H, 4'-CH_3_), 5.44 (s, 2H, 5-H), 5.70–5.81 (m, 2H, 17-H), 6.54 (s, 1H, 20-OH), 7.37 (s, 1H, 14-H), 7.60 (d, *J =* 8.8Hz, 2H, 2', 6'-H), 7.84 (d, *J =* 8.8Hz, 2H, 3', 5'-H), 7.85 (t, 1H, 11-H), 7.95 (t, 1H, 10-H), 8.26 (d, *J =* 8.4 Hz, 1H, 12-H), 8.35 (d, *J =* 8.4 Hz, 1H, 9-H), 9.20 (s, 1H, 7-CH), 10.10 (s, 1H, CSNHC), 12.43 (s, 1H, NNH). Anal. calc. for C_28_H_22_N_5_O_4_SBr: C 55.64%, H 3.67%, N 11.59%. Found: C 55.64%, H 3.65%, N 11.61%.

*7-[N-(4-methoxylphenyl)-thioureidoiminomethyl]-camptothecin* (**4e**). Yield: 82%; mp: 234 °C (decomp.); IR(KBr) cm^−1^: 3437 (OH), 3325, 3262 (N-H), 1728 (γ-lactone), 1653 (C=N), 1251 (C=S); ^1^H-NMR (400 MHz, DMSO-*d*_6_) δ: 0.86–0.89 (m, 3H, 19-H), 1.82–1.92 (m, 2H, 18-H), 3.78 (s, 3H, 4'-OCH_3_), 5.43 (s, 2H, 5-H), 5.64–5.76 (m, 2H, 17-H), 6.54 (s, 1H, 20-OH), 6.97 (d, *J =* 8.8Hz, 1H, 2', 6'-H), 7.36 (s, 1H, 14-H), 7.67 (d, *J =* 8.8Hz, 1H, 3', 5'-H), 7.82 (t, 1H, 11-H), 7.92 (t, 1H, 10-H), 8.23 (d, *J =* 8.4 Hz, 1H, 12-H), 8.32 (d, *J =* 8.4 Hz, 1H, 9-H), 9.15 (s, 1H, 7-CH), 9.91 (s, 1H, CSNHC), 12.22 (s, 1H, NNH). Anal. calc. for C_29_H_25_N_5_O_5_S: C 62.69%, H 4.54%, N 12.60%. Found: C 62.67%, H 4.56%, N 12.60%.

*7-[N-(4-methylphenyl)-thioureidoiminomethyl]-camptothecin* (**4f**). Yield: 73%; mp: 246 °C (decomp.); IR(KBr) cm^−1^: 3439 (OH), 3325, 3284 (N-H), 1731 (γ-lactone), 1650 (C=N), 1250 (C=S); ^1^H-NMR (400 MHz, DMSO-*d*_6_) δ: 0.86–0.89 (m, 3H, 19-H), 1.82–1.93 (m, 2H, 18-H), 2.33 (s, 3H, 4'-CH_3_), 5.44 (s, 2H, 5-H), 5.69–5.81 (m, 2H, 17-H), 6.54 (s, 1H, 20-OH), 7.22 (d, *J =* 8.0Hz, 2H, 2', 6'-H), 7.37 (s, 1H, 14-H), 7.73 (d, *J =* 8.0Hz, 2H, 3', 5'-H), 7.85 (t, 1H, 11-H), 7.94 (t, 1H, 10-H), 8.25 (d, *J =* 8.4 Hz, 1H, 12-H), 8.34 (d, *J =* 8.4 Hz, 1H, 9-H), 9.20 (s, 1H, 7-CH), 9.97 (s, 1H, CSNHC), 12.29 (s, 1H, NNH). Anal. calc. for C_29_H_25_N_5_O_4_S: C 64.55%, H 4.67%, N 12.98%. Found: C 64.56%, H 4.66%, N 12.98%.

*7-[N-(2-methoxylphenyl)-thioureidoiminomethyl]-camptothecin* (**4g**). Yield: 81%; mp: 272 °C (decomp.); IR(KBr) cm^−1^: 3436 (OH), 3324 (N-H), 1741 (γ-lactone), 1657 (C=N), 1233 (C=S); ^1^H-NMR (400 MHz, DMSO-*d*_6_) δ: 0.86–0.90 (m, 3H, 19-H), 1.82–1.93 (m, 2H, 18-H), 3.88 (s, 3H, 2'-OCH_3_), 5.42 (s, 2H, 5-H), 5.53–5.64 (m, 2H, 17-H), 6.53 (s, 1H, 20-OH), 6.98–7.02 (m, 1H, 4'-H), 7.12–7.14 (m, 1H, 3'-H), 7.22–7.24 (m, 1H, 5'-H), 7.37 (s, 1H, 14-H), 7.85 (t, 1H, 11-H), 7.94 (t, 1H, 10-H), 8.25 (d, *J =* 8.4 Hz, 1H, 12-H), 8.38–8.41 (m, 2H, 9, 6' -H), 9.22 (s, 1H, 7-CH), 9.83 (s, 1H, CSNHC), 12.35 (s, 1H, NNH). Anal. calc. for C_29_H_25_N_5_O_5_S: C 62.69%, H 4.54%, N 12.60%. Found: C 62.68%, H 4.55%, N 12.61%.

*7-[N-(2-methylphenyl)-thioureidoiminomethyl]-camptothecin* (**4h**). Yield: 77%; mp: 265 °C (decomp.); IR(KBr) cm^−1^: 3471 (OH), 3279, 3126 (N-H), 1741 (γ-lactone), 1664 (C=N), 1257 (C=S); ^1^H-NMR (400 MHz, DMSO-*d*_6_) δ: 0.85–0.88 (m, 3H, 19-H), 1.82–1.90 (m, 2H, 18-H), 2.38 (s, 3H, 2'-CH_3_), 5.41 (s, 2H, 5-H), 5.66–5.82 (m, 2H, 17-H), 6.53 (s, 1H, 20-OH), 7.22–7.42 (m, 4H, 3', 4', 5', 6'-H), 7.35 (s, 1H, 14-H), 7.83 (t, 1H, 11-H), 7.93 (t, 1H, 10-H), 8.24 (d, *J =* 8.4 Hz, 1H, 12-H), 8.35 (d, *J =* 8.4 Hz, 1H, 9-H), 9.19 (s, 1H, 7-CH), 9.82 (s, 1H, CSNHC), 12.25 (s, 1H, NNH). Anal. calc. for C_29_H_25_N_5_O_4_S: C 64.55%, H 4.67%, N 12.98%. Found: C 64.56%, H 4.65%, N 12.99%.

*7-[N-(1-naphthyl)-thioureidoiminomethyl]-camptothecin* (**4i**). Yield: 87%; mp: 278 °C (decomp.); IR(KBr) cm^−1^: 3436 (broad, OH, N-H), 1745 (γ-lactone), 1656 (C=N), 1227(C=S); ^1^H-NMR (400 MHz, DMSO-*d*_6_) δ: 0.85–0.88 (m, 3H, 19-H), 1.80–1.91 (m, 2H, 18-H), 5.39 (s, 2H, 5-H), 5.74–5.90 (m, 2H, 17-H), 6.53 (s, 1H, 20-OH), 7.36 (s, 1H, 14-H), 7.55–7.66 (m, 4H, naphthyl-H), 7.85 (t, 1H, 11-H), 7.93 (t, 1H, 10-H), 7.94–8.01 (m, 3H, naphthyl-H), 8.25 (d, *J =* 8.4 Hz, 1H, 12-H), 8.42 (d, *J =* 8.4 Hz, 1H, 9-H), 9.23 (s, 1H, 7-CH), 10.37 (s, 1H, CSNHC), 12.37 (s, 1H, NNH). Anal. calc. for C_32_H_25_N_5_O_4_S: C 66.77%, H 4.38%, N 12.17%. Found: C 66.76%, H 4.37%, N 12.19%.

*7-[N-(4-fluorophenyl)-thioureidoiminomethyl]-camptothecin* (**4j**). Yield: 74%; mp: 271 °C (decomp.); IR(KBr) cm^−1^: 3438 (OH), 3321, 3266 (N-H), 1744 (γ-lactone), 1655 (C=N), 1236 (C=S); ^1^H-NMR (400 MHz, DMSO-*d*_6_) δ: 0.87–0.91 (m, 3H, 19-H), 1.85–1.92 (m, 2H, 18-H), 5.45 (s, 2H, 5-H), 5.74–5.81 (m, 2H, 17-H), 6.56 (s, 1H, 20-OH), 7.25–7.29 (m, 2H, 2', 6'-H), 7.38 (s, 1H, 14-H), 7.81–7.85 (m, 2H, 3', 5'-H), 7.87 (s, 1H, 11-H), 7.95 (t, 1H, 10-H), 8.25 (d, *J =* 8.4 Hz, 1H, 12-H), 8.35 (d, *J =* 8.4 Hz, 1H, 9-H), 9.19 (s, 1H, 7-CH), 10.07 (s, 1H, CSNHC), 12.37 (s, 1H, NNH). Anal. calc. for C_28_H_22_N_5_O_4_SF: C 61.87%, H 4.08%, N 12.88%. Found: C 61.88%, H 4.09%, N 12.86%.

*7-[N-(3-chlorinephenyl)-thioureidoiminomethyl]-camptothecin* (**4k**). Yield: 69%; mp: 269 °C (decomp.); IR(KBr) cm^−1^: 3439 (OH), 3267, 3132 (N-H), 1741 (γ-lactone), 1651 (C=N), 1254 (C=S); ^1^H-NMR (400 MHz, DMSO-*d*_6_) δ: 0.87–0.91 (m, 3H, 19-H), 1.86–1.90 (m, 2H, 18-H), 5.45 (s, 2H, 5-H), 5.75–5.77 (m, 2H, 17-H), 6.56 (s, 1H, 20-OH), 7.31 (d, *J =* 7.6 Hz, 1H, 6'-H), 7.38 (s, 1H, 14-H), 7.46 (t, 1H, 5'-H), 7.80 (d, *J =* 9.6 Hz, 1H, 4'-H), 7.86 (t, 1H, 11-H), 7.95 (t, 1H, 10-H), 8.12 (s, 1H, 2'-H), 8.25 (d, *J =* 8.4 Hz, 1H, 12-H), 8.35 (d, *J =* 8.4 Hz, 1H, 9-H), 9.22 (s, 1H, 7-CH), 10.14 (s, 1H, CSNHC), 12.47 (s, 1H, NNH). Anal. calc. for C_28_H_22_N_5_O_4_SCl: C 60.05%, H 3.96%, N 12.51%. Found: C 60.07%, H 3.93%, N 12.52%.

*7-[N-cyclohexyl-thioureidoiminomethyl]-camptothecin* (**4l**). Yield: 58%; mp: 263 °C (decomp.); IR(KBr) cm^−1^: 3438 (OH), 3279, 3126 (N-H), 1746 (γ-lactone), 1652 (C=N), 1226 (C=S); ^1^H-NMR (400 MHz, DMSO-*d*_6_) δ: 0.87–0.91 (m, 3H, 19-H), 1.21–1.27 (m, 1H, cyclohexane-H), 1.32–1.40 (m, 2H, cyclohexane-H), 1.47–1.55 (m, 2H, cyclohexane-H), 1.63–1.65 (m, 1H, cyclohexane-H), 1.77–1.80 (m, 2H, cyclohexane-H), 1.86–1.90 (m, 2H, 18-H), 2.01–2.03 (m, 2H, cyclohexane-H), 4.19 (s, 1H, cyclohexane-H), 5.46 (s, 2H, 5-H), 5.53 (s, 2H, 17-H), 6.56 (s, 1H, 20-OH), 7.36 (s, 1H, 14-H), 7.83 (t, 1H, 11-H), 7.89 (d, *J =* 8.4 Hz, 1H, CSNHC), 7.94 (t, 1H, 10-H), 8.24 (d, *J =* 8.8 Hz, 1H, 12-H), 8.28 (d, *J =* 8.4 Hz, 1H, 9-H), 9.12 (s, 1H, 7-CH), 12.05 (s, 1H, NNH). Anal. calc. for C_28_H_29_N_5_O_4_S: C 63.26%, H 5.50%, N 13.17%. Found: C 63.25%, H 5.51%, N 13.17%.

### 3.3. General Synthetic Procedure for Target Compounds **5a**–**l**

To a solution of 7-formyl camptothecin (0.1 mmol) in chloroform (10 mL) and methanol (10 mL), an appropriate *N*-substituent-carbamidohydrazine (0.12 mmol) and 10% hydrochloric acid (0.5 mL) were added. The reaction mixture was stirred for 2 h at room temperature. A large amount of precipitate was produced. After filtration, the precipitate was washed with chloroform to give a pale yellow solid. Recrystallization from N, N-dimethyl dimethylformamide gave compounds **5a**–**l**.

*7-(N-phenylaminocarbonylhydrazonomethyl)camptothecin* (**5a**). Yield: 82%; mp: 232 °C (decomp.); IR(KBr) cm^−1^: 3397 (broad, OH, N-H), 1751 (γ-lactone), 1689 (C=O) 1658(C=N); ^1^H-NMR (400 MHz, DMSO-*d*_6_) δ: 0.87–0.91 (m, 3H, 19-H), 1.83–1.93 (m, 2H, 18-H), 5.45 (s, 2H, 5-H), 5.67 (s, 2H, 17-H), 6.53 (s, 1H, 20-OH), 7.05–7.08 (m, 1H, 4'-H), 7.33–7.35 (m, 2H, 2', 6'-H), 7.36 (s, 1H, 14-H), 7.75–7.77 (m, 2H, 3', 5'-H), 7.80 (t, 1H, 11-H), 7.90 (t, 1H, 10-H), 8.22 (d, *J =* 8.4 Hz, 1H, 12-H), 8.34 (d, *J =* 8.4 Hz, 1H, 9-H), 8.79 (s, 1H, 7-CH), 8.96 (s, 1H, CONHC), 11.37 (s, 1H, NNH). Anal. alc. for C_28_H_23_N_5_O_5_: C 66.00%, H 4.55%, N 13.75%. Found: C 66.02%, H 4.53%, N 13.75%.

*7-[N-(4-fluorophenyl)-carbonylhydrazonomethyl]-camptothecin* (**5b**). Yield: 74%; mp: 236 °C (decomp.); IR(KBr) cm^−1^: 3406 (broad, OH, N-H), 1742 (γ-lactone), 1706 (C=O), 1656 (C=N); ^1^H-NMR (400 MHz, DMSO-*d*_6_) δ: 0.87–0.90 (m, 3H, 19-H), 1.82–1.93 (m, 2H, 18-H), 5.46 (s, 2H, 5-H), 5.71 (s, 2H, 17-H), 6.55 (s, 1H, 20-OH), 7.17–7.21 (m, 2H, 2', 6'-H), 7.37 (s, 1H, 14-H), 7.75–7.78 (m, 2H, 3', 5'-H), 7.83 (t, 1H, 11-H), 7.93 (t, 1H, 10-H), 8.24 (d, *J =* 8.0 Hz, 1H, 12-H), 8.36 (d, *J =* 8.0 Hz, 1H, 9-H), 8.92 (s, 1H, 7-CH), 8.98 (s, 1H, CONHC), 11.40 (s, 1H, NNH). Anal. calc. for C_28_H_22_N_5_O_5_F: C 63.75%, H 4.20%, N 13.28%. Found: C 63.75%, H 4.21%, N 13.27%.

*7-[N-(4-chlorophenyl)-carbonylhydrazonomethyl]-camptothecin* (**5c**). Yield: 85%; mp: 225 °C (decomp.); IR(KBr) cm^−1^: 3294–3401 (broad, OH, N-H), 1751 (γ-lactone), 1706 (C=O), 1668 (C=N); ^1^H-NMR (400 MHz, DMSO-*d*_6_) δ: 0.87–0.91 (m, 3H, 19-H), 1.82–1.93 (m, 2H, 18-H), 5.46 (s, 2H, 5-H), 5.65 (s, 2H, 17-H), 6.53 (s, 1H, 20-OH), 7.36 (s, 1H, 14-H), 7.39 (d, *J =* 8.8Hz, 2H, 2', 6'-H), 7.79 (d, *J =* 8.8Hz, 2H, 3', 5'-H), 7.80 (t, 1H, 11-H), 7.91 (t, 1H, 10-H), 8.22 (d, *J =* 8.4 Hz, 1H, 12-H), 8.34 (d, *J =* 8.4 Hz, 1H, 9-H), 8.96 (s, 1H, 7-CH), 8.97 (s, 1H, CONHC), 11.43 (s, 1H, NNH). Anal. calc. for C_28_H_22_N_5_O_5_Cl: C 61.82%, H 4.08%, N 12.87%. Found: C 61.81%, H 4.09%, N 12.87%.

*7-[N-(4-bromophenyl)-carbonylhydrazonomethyl]-camptothecin* (**5d**). Yield: 72%; mp: 237 °C (decomp.); IR(KBr) cm^−1^: 3443 (OH), 3393 (N-H), 1733 (γ-lactone), 1709 (C=O), 1650 (C=N); ^1^H-NMR (400 MHz, DMSO-*d*_6_) δ: 0.87–0.91 (m, 3H, 19-H), 1.83–1.93 (m, 2H, 18-H), 5.45 (s, 2H, 5-H), 5.66 (s, 2H, 17-H), 6.54 (s, 1H, 20-OH), 7.36 (s, 1H, 14-H), 7.51 (d, *J =* 8.8Hz, 2H, 2', 6'-H), 7.74 (d, *J =* 8.8Hz, 2H, 3', 5'-H), 7.80 (t, 1H, 11-H), 7.91 (t, 1H, 10-H), 8.22 (d, *J =* 8.4 Hz, 1H, 12-H), 8.34 (d, *J =* 8.4 Hz, 1H, 9-H), 8.96 (s, 1H, 7-CH), 8.97 (s, 1H, CONHC), 11.45 (s, 1H, NNH). Anal. calc. for C_28_H_22_N_5_O_5_Br: C 57.15%, H 3.77%, N 11.90%. Found: C 57.15%, H 3.76%, N 11.91%.

*7-[N-(4-methoxyphenyl)-carbonylhydrazonomethyl]-camptothecin* (**5e**). Yield: 76%; mp: 254 °C (decomp.); IR(KBr) cm^−1^: 3259–3409 (broad, OH, N-H), 1739 (γ-lactone), 1687 (C=O), 1661 (C=N); ^1^H-NMR (400 MHz, DMSO-*d*_6_) δ: 0.87–0.90 (m, 3H, 19-H), 1.83–1.93 (m, 2H, 18-H), 3.75(s, 3H, 4'-OCH_3_), 5.46 (s, 2H, 5-H), 5.67–5.70 (m, 2H, 17-H), 6.54 (s, 1H, 20-OH), 6.92 (d, *J =* 8.8Hz, 2H, 2', 6'-H), 7.37 (s, 1H, 14-H), 7.65 (d, *J =* 8.8Hz, 2H, 3', 5'-H), 7.82 (t, 1H, 11-H), 7.92 (t, 1H, 10-H), 8.23 (d, *J =* 8.4 Hz, 1H, 12-H), 8.35 (d, *J =* 8.4 Hz, 1H, 9-H), 8.70 (s, 1H, 7-CH), 8.96 (s, 1H, CONHC), 11.31 (s, 1H, NNH). Anal. calc. for C_29_H_25_N_5_O_6_: C 64.56%, H 4.67%, N 12.98%. Found: C 64.56%, H 4.68%, N 12.97%.

*7-[N-(4-methylphenyl)-carbonylhydrazonomethyl]-camptothecin* (**5f**). Yield: 87%; mp: 239 °C (decomp.); IR(KBr) cm^−1^: 3502 (OH), 3401, 3211(N-H), 1749 (γ-lactone), 1688 (C=O), 1659 (C=N); ^1^H-NMR (400 MHz, DMSO-*d*_6_) δ: 0.87–0.91 (m, 3H, 19-H), 1.83–1.93 (m, 2H, 18-H), 2.28(s, 3H, 4'-CH_3_), 5.46 (s, 2H, 5-H), 5.66 (s, 2H, 17-H), 6.54 (s, 1H, 20-OH), 7.15 (d, *J =* 8.4Hz, 2H, 2', 6'-H), 7.36 (s, 1H, 14-H), 7.64 (d, *J =* 8.4Hz, 2H, 3', 5'-H), 7.79–7.83 (m, 1H, 11-H), 7.89–7.93 (m, 1H, 10-H), 8.22 (d, *J =* 8.0 Hz, 1H, 12-H), 8.34 (d, *J =* 8.0 Hz, 1H, 9-H), 8.71 (s, 1H, 7-CH), 8.96 (s, 1H, CONHC), 11.35 (s, 1H, NNH). Anal. calc. for C_29_H_25_N_5_O_5_: C 66.53%, H 4.81%, N 13.38%. Found: C 66.52%, H 4.81%, N 13.37%.

*7-[N-(2-methoxyphenyl)-carbonylhydrazonomethyl]-camptothecin* (**5g**). Yield: 78%; mp: 242 °C (decomp.); IR(KBr) cm^−1^: 3469 (OH), 3384, 3298 (N-H), 1746 (γ-lactone), 1686 (C=O), 1660 (C=N); ^1^H-NMR (400 MHz, DMSO-*d*_6_) δ: 0.87–0.91 (m, 3H, 19-H), 1.86–1.90 (m, 2H, 18-H), 3.91(s, 3H, 2'-OCH_3_), 5.44 (s, 2H, 5-H), 5.51 (s, 2H, 17-H), 6.54 (s, 1H, 20-OH), 6.93–7.09 (m, 3H, 3', 4', 5'-H), 7.37 (s, 1H, 14-H), 7.83 (t, 1H, 11-H), 7.93 (t, 1H, 10-H), 8.17(dd, *J =* 8.0, 1.2Hz, 6'-H), 8.25 (d, *J =* 8.4 Hz, 1H, 12-H), 8.42 (d, *J =* 8.4 Hz, 1H, 9-H), 8.69 (s, 1H, 7-CH), 9.00 (s, 1H, CONHC), 11.47 (s, 1H, NNH). Anal. calc. for C_29_H_25_N_5_O_6_: C 64.56%, H 4.67%, N 12.98%. Found: C 64.54%, H 4.68%, N 12.98%.

*7-[N-(3-bromophenyl)-carbonylhydrazonomethyl]-camptothecin* (**5h**). Yield: 72%; mp: 253 °C (decomp.); IR(KBr) cm^−1^: 3282–3396 (broad, OH, N-H), 1746 (γ-lactone), 1699 (C=O), 1665 (C=N); ^1^H-NMR (400 MHz, DMSO-*d*_6_) δ: 0.87–0.90 (m, 3H, 19-H), 1.82–1.93 (m, 2H, 18-H), 5.46 (s, 2H, 5-H), 5.70 (s, 2H, 17-H), 6.55 (s, 1H, 20-OH), 7.11–7.33 (m, 3H, 4', 5', 6'-H), 7.36 (s, 1H, 14-H), 7.70 (d, *J =* 8.4Hz, 1H, 2'-H), 7.81–7.86 (m, 1H, 11-H), 7.93 (t, 1H, 10-H), 8.24 (d, *J =* 8.4 Hz, 1H, 12-H), 8.36 (d, *J =* 8.4 Hz, 1H, 9-H), 8.99 (s, 1H, 7-CH), 9.04 (s, 1H, CONHC), 11.49 (s, 1H, NNH). Anal. calc. for C_28_H_22_N_5_O_5_Br: C 57.15%, H 3.77%, N 11.90%. Found: C 57.16%, H 3.76%, N 11.92%.

*7-[N-(2-fluorophenyl)-carbonylhydrazonomethyl]-camptothecin* (**5i**). Yield: 67%; mp: 232 °C (decomp.); IR(KBr) cm^−1^: 3276–3398 (broad, OH, N-H), 1732 (γ-lactone), 1702 (C=O), 1655 (C=N); ^1^H-NMR (400 MHz, DMSO-*d*_6_) δ: 0.86–0.90 (m, 3H, 19-H), 1.85–1.89 (m, 2H, 18-H), 5.43 (s, 2H, 5-H), 5.54 (s, 2H, 17-H), 6.54 (s, 1H, 20-OH), 7.15–7.34 (m, 3H, 3', 4', 5'-H), 7.35 (s, 1H, 14-H), 7.80–7.84 (m, 1H, 6'-H), 7.90–7.92 (m, 1H, 11-H), 7.94–8.00 (m, 1H, 10-H), 8.23 (d, *J =* 8.0 Hz, 1H, 12-H), 8.43 (d, *J =* 8.0 Hz, 1H, 9-H), 8.68 (s, 1H, 7-CH), 8.97 (s, 1H, CONHC), 11.45 (s, 1H, NNH). Anal. calc. for C_28_H_22_N_5_O_5_F: C 63.75%, H 4.20%, N 13.28%. Found: C 63.74%, H 4.23%, N 13.26%.

*7-[N-(3-chlorophenyl)-carbonylhydrazonomethyl]-camptothecin* (**5j**). Yield: 83%; mp: 248 °C (decomp.); IR(KBr) cm^−1^: 3631 (OH), 3398, 3292 (N-H), 1751 (γ-lactone), 1691(C=O), 1659 (C=N); ^1^H-NMR (400 MHz, DMSO-*d*_6_) δ: 0.87–0.90 (m, 3H, 19-H), 1.82–1.93 (m, 2H, 18-H), 5.45 (s, 2H, 5-H), 5.69 (s, 2H, 17-H), 6.55 (s, 1H, 20-OH), 7.11(dd, *J =* 8.0, 1.2Hz, 1H, 6'-H), 7.34–7.38 (m, 1H, 5'-H), 7.37 (s, 1H, 14-H), 7.65–7.67 (m, 1H, 4'-H), 7.82 (t, 1H, 11-H), 7.89 (t, 1H, 10-H), 7.94–7.96 (m, 1H, 2'-H), 8.23 (d, *J =* 8.4 Hz, 1H, 12-H), 8.35 (d, *J =* 8.4 Hz, 1H, 9-H), 8.98 (s, 1H, 7-CH), 9.03 (s, 1H, CONHC), 11.49 (s, 1H, NNH). Anal. calc. for C_28_H_22_N_5_O_5_Cl: C 61.82%, H 4.08%, N 12.87%. Found: C 61.84%, H 4.07%, N 12.85%.

*7-[N-(1-naphthyl)-carbonylhydrazonomethyl]-camptothecin* (**5k**). Yield: 84%; mp: 275 °C (decomp.); IR(KBr) cm^−1^: 3402 (broad, OH, N-H), 1741 (γ-lactone), 1694 (C=O), 1658 (C=N); ^1^H-NMR (400 MHz, DMSO-*d*_6_) δ: 0.88–0.91 (m, 3H, 19-H), 1.84–1.98 (m, 2H, 18-H), 5.50 (s, 2H, 5-H), 5.70–5.80 (m, 2H, 17-H), 6.53 (s, 1H, 20-OH), 7.38 (s, 1H, 14-H), 7.38–7.50 (m, 2H, naphthyl-H), 7.83 (t, 1H, 11-H), 7.85–7.90 (m, 4H, naphthyl-H), 7.93 (t, 1H, 10-H), 8.24 (d, *J =* 8.0 Hz, 1H, 12-H), 8.36 (d, *J =* 8.4 Hz, 1H, 9-H), 9.01 (s, 1H, 7-CH), 9.03 (s, 1H, CONHC), 11.54 (s, 1H, NNH). Anal. calc. for C_32_H_25_N_5_O_5_: C 68.69%, H 4.50%, N 12.52%. Found: C 68.69%, H 4.51%, N 12.54%.

*7-[N-(2-chlorophenyl)-carbonylhydrazonomethyl]-camptothecin* (**5l**). Yield: 69%; mp: 251 °C (decomp.); IR(KBr) cm^−1^: 3281–3397 (broad, OH, N-H), 1748 (γ-lactone), 1689 (C=O), 1664 (C=N); ^1^H-NMR (400 MHz, DMSO-*d*_6_) δ: 0.88–0.92 (m, 3H, 19-H), 1.87–1.90 (m, 2H, 18-H), 5.45 (s, 2H, 5-H), 5.56 (s, 2H, 17-H), 6.55 (s, 1H, 20-OH), 7.13 (t, 1H, 4'-H), 7.37 (s, 1H, 14-H), 7.38 (t, 1H, 5'-H), 7.56 (d, *J =* 8.0 Hz, 1H, 3'-H), 7.83 (t, 1H, 11-H), 7.93 (t, 1H, 10-H), 8.27(dd, *J =* 8.0, 18.0 Hz, 6'-H), 8.32 (d, *J =* 8.4 Hz, 1H, 12-H), 8.40 (d, *J =* 8.4 Hz, 1H, 9-H), 8.73 (s, 1H, 7-CH), 9.08 (s, 1H, CONHC), 11.58 (s, 1H, NNH). Anal. calc. for C_28_H_22_N_5_O_5_Cl: C 61.82%, H 4.08%, N 12.87%. Found: C 61.83%, H 4.07%, N 12.86%.

### 3.4. General Synthetic Procedure for Target Compounds **6a**–**d**

To a solution of 7-formyl camptothecin (0.1 mmol) in chloroform (10 mL) and methanol (10 mL), an appropriate *N*-acylsubstituent-thioureidohydrazine (0.12 mmol) and 10% hydrochloric acid (0.5 mL) were added. The reaction mixture was stirred for 2 h at room temperature. A large amount of precipitate was produced. After filtration, the precipitate was washed with chloroform to give a deep yellow solid. Recrystallization from N, N-dimethyl dimethylformamide gave compounds **6a**–**d**.

*7-(N-benzoylthioureidoiminomethyl)camptothecin* (**6a**). Yield: 91%; mp: 277 °C (decomp.); IR(KBr) cm^−1^: 3435 (broad, OH, N-H), 1745 (γ-lactone), 1660 (C=O), 1602 (C=N), 1229 (C=S); ^1^H-NMR (400 MHz, DMSO-*d*_6_) δ: 0.87–0.91 (m, 3H, 19-H), 1.84–1.91 (m, 2H, 18-H), 4.89 (d, *J =* 5.6 Hz, 2H, benzyl-CH_2_), 5.44 (s, 2H, 5-H), 5.54 (s, 2H, 17-H), 6.54 (s, 1H, 20-OH), 7.36 (s, 1H, 14-H), 7.55–7.59 (m, 2H, 2', 6'-H), 7.67–7.71 (m, 1H, 4'-H), 7.83 (t, 1H, 11-H), 7.92 (t, 1H, 10-H), 8.03–8.05 (m, 2H, 3', 5'-H), 8.26 (d, *J =* 8.4 Hz, 1H, 12-H), 8.76 (d, *J =* 8.4 Hz, 1H, 9-H), 9.81 (s, 1H, 7-CH), 11.94 (s, 1H, CSNHCO), 13.99(s, 1H, NNH). Anal. calc. cor C_29_H_23_N_5_O_5_S: C 62.92%, H 4.19%, N 12.65%. Found: C 62.91%, H 4.18%, N 12.67%.

*7-[N-(4-methylbenzoyl)-thioureidoiminomethyl]-camptothecin* (**6b**). Yield: 84%; mp: 282 °C (decomp.); IR(KBr) cm^−1^: 3437 (broad, OH, N-H), 1744 (γ-lactone), 1658 (C=O), 1598 (C=N), 1229 (C=S); ^1^H-NMR (400 MHz, DMSO-*d*_6_) δ: 0.88–0.92 (m, 3H, 19-H), 1.84–1.95 (m, 2H, 18-H), 2.35(s, 3H, 4'-CH_3_), 5.46 (s, 2H, 5-H), 5.69–5.83 (m, 2H, 17-H), 6.56 (s, 1H, 20-OH), 7.24 (d, *J =* 8.0Hz, 2H, 2', 6'-H), 7.39 (s, 1H, 14-H), 7.75 (d, *J =* 8.0Hz, 2H, 3', 5'-H), 7.87 (t, 1H, 11-H), 7.96 (t, 1H, 10-H), 8.27 (d, *J =* 8.4 Hz, 1H, 12-H), 8.36 (d, *J =* 8.4 Hz, 1H, 9-H), 9.22 (s, 1H, 7-CH), 9.99 (s, 1H, CSNHCO), 12.31 (s, 1H, NNH). Anal. calc. for C_30_H_25_N_5_O_5_S: C 63.48%, H 4.44%, N 12.34%. Found: C 63.47%, H 4.43%, N 12.35%.

*7-[N-(4chlorobenzoyl)-thioureidoiminomethyl]-camptothecin* (**6c**). Yield: 76%; mp: 269 °C (decomp.); IR(KBr) cm^−1^: 3438 (broad, OH, N-H), 1746 (γ-lactone), 1658 (C=O), 1599 (C=N), 1248 (C=S); ^1^H-NMR (400 MHz, DMSO-*d*_6_) δ: 0.87–0.91 (m, 3H, 19-H), 1.82–1.94 (m, 2H, 18-H), 5.51 (s, 2H, 5-H), 5.76–5.77 (m, 2H, 17-H), 6.56 (s, 1H, 20-OH), 7.38 (s, 1H, 14-H), 7.48 (d, *J =* 8.8Hz, 2H, 2', 6'-H), 7.84–7.88 (m, 1H, 11-H), 7.92 (d, *J =* 8.8Hz, 2H, 3', 5'-H), 7.95–7.97 (m, 1H, 10-H), 8.27 (d, *J =* 8.0 Hz, 1H, 12-H), 8.36 (d, *J =* 8.0 Hz, 1H, 9-H), 9.21 (s, 1H, 7-CH), 10.12 (s, 1H, CSNHCO), 12.43 (s, 1H, NNH). Anal. calc. for C_29_H_22_N_5_O_5_SCl: C 59.23%, H 3.77%, N 11.91%. Found: C 59.25%, H 3.76%, N 11.93%.

*7-[N-(2-naphthoyl)-thioureidoiminomethyl] -camptothecin* (**6d**). Yield: 77%; mp: 275 °C (decomp.); IR(KBr) cm^−1^: 3439 (broad, OH, N-H), 1747 (γ-lactone), 1658 (C=O), 1598 (C=N), 1228 (C=S); ^1^H-NMR (400 MHz, DMSO-*d*_6_) δ: 0.85–0.89 (m, 3H, 19-H), 1.81–1.92 (m, 2H, 18-H), 5.40 (s, 2H, 5-H), 5.75–5.91 (m, 2H, 17-H), 6.54 (s, 1H, 20-OH), 7.37 (s, 1H, 14-H), 7.56–7.67 (m, 4H, naphthyl-H), 7.86 (t, 1H, 11-H), 7.94 (t, 1H, 10-H), 7.95–8.02 (m, 3H, naphthyl-H), 8.26 (d, *J =* 8.4 Hz, 1H, 12-H), 8.43 (d, *J =* 8.4 Hz, 1H, 9-H), 9.24 (s, 1H, 7-CH), 10.38 (s, 1H, CSNHCO), 12.38 (s, 1H, NNH). Anal. calc. for C_33_H_25_N_5_O_5_S: C 65.66%, H 4.17%, N 11.60%. Found: C 65.67%, H 4.16%, N 11.61%.

### 3.5. Biological Assay

All bioassays were performed on representative test organisms reared in the laboratory. The bioassay was repeated at 25 ± 2 °C according to statistical requirements. Assessments were made on a dead/alive basis, and mortality rates were corrected using Abbott’s formula [39]. Evaluations are based on a percentage scale of 0~100, in which 0 = no activity and 100 = total kill. The deviation of values was ±5%. Probit analysis was used to determine lethal concentrations of 50% (LC_50_) by using the SPSS program, version 13.0. For comparative purposes, CPT was tested as a reference. All bioassay results are summarized in Table 1, Table 2 and Table 3.

#### 3.5.1. Lethal Activity against *Tetranychus cinnabarinus*

The acaricidal activity of compounds **4a**–**l**, **5a**–**l**, **6a**–**d**, and CPT (positive control) was evaluated using the slide immersion method recommended by FAO [40]. Thirty adult spider mites were fixed dorsally to a strip of double-sided tape attached to the slide by using a small brush. The slide was immersed and shaken for 3 s in the diluted solution of the test compound. After the excess solution was removed, the treated slides with the mites were kept at 25 ± 2 °C in a Petri dish with moist filter paper. Mortality rates were calculated 24 h after treatment. Each treatment was replicated with triplicate experiments and each replicate involved 30 adult mites. Control groups were tested with acetone only.

#### 3.5.2. Lethal Activity against *Brevicoryne brassicae*

The insecticidal activity of compounds **4a**–**l**, **5a**–**l**, **6a**–**d**, and CPT (positive control) against *B. brassicae* was evaluated according to the reported procedure [41]. Thirty healthy adult aphids were dipped into the diluted solutions of tested compound for 5 s, superfluous fluid was removed, and aphids were placed in an air-conditioned room. Mortality rates were calculated 24 h after treatment. Each treatment was performed in triplicate. Control groups were tested with acetone only.

#### 3.5.3. Lethal Activity against *Bursaphelenchus xylophilus*

Acetone solutions of compounds **4a**–**l**, **5a**–**l**, **6a**–**d**, and CPT (positive control) were first prepared at different concentrations. Then 10 μL of the above solutions was added to the aqueous suspension (90 μL) containing approximately 2500 living nematodes (third-instar and fourth-instar larvae of B. xylophilus) per milliliter. The blank control group was prepared in the same way but lacked the tested compound. Three replicates in each trial were made and kept at 25 °C for 24 h. Finally, the activities of five concentrations of the tested compounds were monitored under a microscope by recording the death rate of the tested nematodes. Nematodes that did not move when prodded with a needle were considered to be dead. The LC_50_ values of tested compounds were calculated using the probit method [42].

### 3.6. CoMFA Analysis

To gain insights into the key structural factors affecting bioactivity, a 3D-QSAR model using comparative molecular field analysis (CoMFA) was performed using the SYBYL 6.9 software package [43]. Firstly, all compounds were sketched. Then, these compounds were minimized using the Tripos force field with a distance-dependent dielectric and Powell conjugate gradient algorithm with a convergence criterion of 0.05 kcal/mol. Partial atomic charges were calculated using the Gasteiger–Hückel charge. Further conformational search was performed with the multisearch routine.

All the molecules were aligned using compound **4d** (the most active compound) as the template. The DATABASE ALIGNMENT method was used to align these molecules to the template with common atoms (in total, 28 atoms) in compound **4d** (Figure 4a). The resulting alignment is shown in Figure 4b. To build and validate the CoMFA model, the dataset was split into training and test sets by considering the distribution of activity of compounds. The training set contained 23 compounds. The test set was composed of five compounds (labeled in Table 3).

After the alignment of compounds, the molecular fields including steric (Lennard–Jones potentials) and electrostatic (Coulomb potentials) field energies were calculated using sp^3^ carbon as a probe atom with a grid step size of 2 Å. The cutoff value of 30 kcal/mol was adopted.

To build the relationship between the molecular fields and the biological activities (*Tetranychus cinnabarinus*), partial least squares (PLS) regression analyses were used to develop CoMFA models using the standard implementation in the SYBYL package. The CoMFA descriptors were used as independent variables, and pLC_50_ values were used as the target variables. Cross-validation in PLS was carried out using the leave-one-out method to obtain the optimal number of components. The final model was constructed with the optimum number of components equal to that yielding the highest *q*^2^. In addition, a test set of molecules with known biological activities that were not included in the model generation was used to further validate the obtained models.

## 4. Conclusions

In summary, three novel series of 7-modified camptothecin derivatives containing thiourea, urea, and acylthiourea moieties were synthesized, and their lethal activity against *T. Cinnabarinus*, *B. xylophilus*, and *B. brassicae* (Linnaeus) was evaluated. Most of the target compounds possessed good-to-excellent activities against three insect species, some of which were much better in comparison with CPT. As we envisioned, SAR analysis indicated an important role of various C-7 modified derivatives in the activity profiles of **1** analogs, and the size, electron density, and distribution of the substituents within the different side chain are critical to the derivatives’ activity. Furthermore, 3D-QSAR model of these derivatives is developed to understand the key structural factors affecting the inhibitory activity. The high predictive ability of the model indicates that it can be reliably used to predict activity of the inhibitors and design novel compounds. In addition, the QSAR analysis indicates that the size of the substituents plays an important role in the activity of 7-modified camptothecin derivatives. This finding indicated that the incorporation of the active fragment into the C-7 position could improve the lethal activity of CPTs as expected and warrant further studies for designing new molecules with good biological activity. Continuing studies to substantiate and improve activity profiles are underway in our laboratory and will be reported in due course.
